# Follow‐up on the U.S. Central Intelligence Agency's (CIA) remote viewing experiments^☆^

**DOI:** 10.1002/brb3.3026

**Published:** 2023-05-03

**Authors:** Álex Escolà‐Gascón, James Houran, Neil Dagnall, Kenneth Drinkwater, Andrew Denovan

**Affiliations:** ^1^ Area of Applied Mathematics and Statistics Ramon Llull University (Blanquerna Foundation) Barcelona Spain; ^2^ Laboratory for Statistics and Computation ISLA—Instituto Politécnico de Gestão e Tecnologia Vila Nova de Gaia Portugal; ^3^ Integrated Knowledge Systems Dallas Texas USA; ^4^ Psychology Department, Faculty of Health, Psychology and Social Care Manchester Metropolitan University Manchester UK; ^5^ Department of People and Performance Faculty of Business and Law Manchester Metropolitan University Manchester UK

**Keywords:** anomalous cognitions, central intelligence agency, emotional intelligence, psi, remote viewing

## Abstract

**Objectives:**

Since 1972, the U.S. Central Intelligence Agency (CIA) commissioned several research programs on remote viewing (RV) that were progressively declassified from 1995 to 2003. The main objectives of this research were to statistically replicate the original findings and address the question: What are the underlying cognitive mechanisms involved in RV? The research focused on emotional intelligence (EI) theory and intuitive information processing as possible hypothetical mechanisms.

**Methods:**

We used a quasi‐experimental design with new statistical control techniques based on structural equation modeling, analysis of invariance, and forced‐choice experiments to accurately objectify results. We measured emotional intelligence with the *Mayer—Salovey–Caruso Emotional Intelligence Test*. A total of 347 participants who were nonbelievers in psychic experiences completed an RV experiment using targets based on location coordinates. A total of 287 participants reported beliefs in psychic experiences and completed another RV experiment using targets based on images of places. Moreover, we divided the total sample into further subsamples for the purpose of replicating the findings and also used different thresholds on standard deviations to test for variation in effect sizes. The hit rates on the psi‐RV task were contrasted with the estimated chance.

**Results:**

The results of our first group analysis were nonsignificant, but the analysis applied to the second group produced significant RV‐related effects corresponding to the positive influence of EI (i.e., hits in the RV experiments were 19.5% predicted from EI) with small to moderate effect sizes (between 0. 457 and 0.853).

**Conclusions:**

These findings have profound implications for a new hypothesis of anomalous cognitions relative to RV protocols. Emotions perceived during RV sessions may play an important role in the production of anomalous cognitions. We propose the *Production‐Identification‐Comprehension* (PIC) emotional model as a function of behavior that could enhance VR test success.

## Graphical abstract

Since 1972, the U.S. Central Intelligence Agency (CIA) commissioned several research programs on remote viewing (RV) that were progressively declassified from 1995 to 2003. We offer new statistical evidence suggesting a new approach to RV research based on the role of emotions and emotional intelligence.

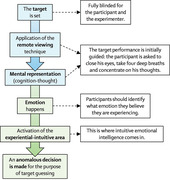

## 1 INTRODUCTION

In 1995, U.S. President Clinton, by order number 1995‐4‐17 entitled “Classified National Security Information,” declassified several research programs (among other contents) funded by the *Central Intelligence Agency* (CIA) and *Defense Intelligence Agency* (DIA) of the United States (Puthoff, 1996). These covert programs were developed over more than 20 years at the *Stanford Research Institute* (SRI, now SRI International) and the *Science Applications International Corporation* (SAIC) (cf. Srinivasan, 2002). Programs addressed remote viewing (RV), that is, determined whether certain individuals, under conditions of perceptual isolation, could access information about places, buildings, photographs, etc., from a distance using putative psi rather than conventional sensory channels (Targ, 2019). The specific objective was to explore whether RV phenomena had enough consistency and stability for use in military espionage (McMoneagle, 2015; Puthoff, 1996). Due to the Cold War and ensuing political‐military tensions between the United States and the former Soviet Union, American Congress classified these programs in the interests of national security (Targ, 1996). The fact that the RV experiments were hidden or classified undermined transparency in scientific research practices. Specifically, other laboratories were not given access to information and were unable to evaluate outcomes with proper methodological or statistical rigor (see the critique by Hyman, 1996 and Nelson et al., 1996).

### 1.1 What is remote viewing?

RV is an experiential technique for altered‐anomalous states (see Utts, 1995, 1996, 1995, 1996, 2018) that allows two types of anomalous cognitions to be subjected to empirical scrutiny (see also Schooler et al., 2018): (a) *precognition* (also called *anticipation of unpredictable stimuli* or *anomalous anticipation of information*, Mossbridge et al., 2012) can be defined as the process by which a person accesses information about the future (i.e., events that have not yet happened) without using sensory or otherwise rational channels recognized by conventional scientific theory (Bem, 2011); and (b) *retro‐cognition* (also called *anomalous information reception* or *clairvoyance*) is defined as the process by which a person accesses content referring to the past (i.e., content that has already happened) without using the conventional channels of biology or logic per current scientific theory (Marwaha & May, 2016). The expression *psi phenomena* or *psi* is a hypothetical construct that has the same definition attributed to anomalous cognitions. However, the term anomalous cognitions is a more neutral label, as the term *psi* is often used by parapsychologists. All these concepts have been sharply criticized on methodological, statistical, or conceptual grounds (e.g., Escolà‐Gascón, 2022a; Houran et al., 2018; Reber & Alcock, 2020; Wagenmakers et al., 2011).

In RV, the participant is asked to visualize the information they intend to access (from the past or the future) (Roe et al., 2020). Then, the participant must mentally and nonverbally represent the distant target or targets to be guessed (May et al., 2011; Scott, 1988). The target is often a specific place, person, or fact (May, 1996; Puthoff, 1996; Targ, 1996). The targets of RV experiments (published in *Nature*, see Targ & Puthoff, 1974) contained specific meanings of interest to U.S. national security (e.g., the location of a secret military base) (see Utts, 1995, 1996, 1995, 1996, 2018). The present study focused on RV relative to *anomalous information reception*, as it is one of the most researched anomalous phenomena showing significant results (see Bem et al., 2016; Tressoldi & Storm, 2021). Unfortunately, the abbreviation for *anomalous information reception* (AIR) is the same as the abbreviation for the *American Institutes for Research* (also AIR) and we wish to prevent confusion. So, henceforth, we use the terms *anomalous cognitions* and RV to refer exclusively to *anomalous information reception*.

### 1.2 Scientific reviews and conclusions after the CIA declassification

Reports on the declassified SRI and SAIC experiments were evaluated in 1995 by statisticians Utts (1995, 1996, 2018) and Hyman (1996) for the *American Institutes for Research*. Although the two authorities agreed on some points, they conflicted on several, with the most significant disagreement being the ultimate conclusions. Utts determined that the evidence from the SRI and SAIC experiments was sufficiently consistent to accept that RV phenomena were empirically validated. In contrast, Hyman did not consider this evidence adequate, criticized some of the methodological procedures applied by SRI, and contended that it did not support the assertion that RV phenomena were “scientifically established.” However, they both agreed on a critical interpretation—namely, that the effect sizes of the experiments conducted at SAIC (which were the most rigorous and addressed methodological problems evident in research conducted at SRI in May, 1996) were consistent and homogeneous. In the words of Hyman (1996, p. 52), *“At best, the results of the SAIC experiments combined with other contemporary findings offer hope that the parapsychologists may be getting closer to the day when they can put something before the scientific community and challenge it to provide an explanation.”* This assertion invited further studies of RV that attempted to replicate the observed effects (see Marwaha & May, 2015).

### 1.3 Subsequent research

Numerous experiments on anomalous cognitions have yielded results statistically favorable (see the original experiments of Maier et al., 2014) and unfavorable (see the replication of Ritchie et al., 2012) to the psi hypothesis. In the case of RV, experiments with significant results greatly predominate (e.g., see another *Nature* publication, Tart et al., 1980, and the contributions of Dunne & Jahn, 2007; Roe et al., 2020; Schmidt et al., 2019) over unsuccessful statistical replications (e.g., Escolà‐Gascón, 2022a; Marks & Kammann, 1978).

A curious trend and one that should be considered in this context are *sheep‐goat* effects. In this effect, individuals who are advocates of parapsychology and who have had psi experiences tend to get a higher number of hits than non‐psi experiencers (Thalbourne, 2001; Thalbourne & Houran, 2003; Thalbourne & Storm, 2012). This trend was obtained even in unsuccessful psi replications recently published (e.g., Escolà‐Gascón, 2022a). Although it is not known why this effect occurs, some evidence suggests that it may be a bias related to response repetition (e.g., Brugger et al., 1990); in any case, the distinction between believers and nonbelievers is supported by evidence and is appropriate to apply.

Researchers addressing these issues are positioned in two groups with conflicting stances: (a) one group includes scientists advocating RV and anomalous cognitions (due to the cumulative empirical evidence, e.g., Cardeña, 2018); and (b) the other group of researchers who are currently not persuaded by the significant evidence for anomalous cognitions and, due to other replications without statistical successes, reject the validity of putative psi (e.g., Reber & Alcock, 2020). Although both positions have empirical support (Escolà‐Gascón, 2020a, 2020b; Escolà‐Gascón et al., 2021), the current issue for these groups is the ideological radicalization they have undergone in the last few decades (Carter, 2011; Leiter, 2002). This extreme scientific prejudice resulted in the marginalization of RV and the scientific study of anomalous cognitions (e.g., Odling‐Smee, 2007).

Other researchers, who are more neutral to these polarized ideologies, have emphasized the need for more research because the statistical evidence to date is insufficient due to the extraordinary epistemic characteristics of RV phenomena (see Hyman, 1996). Moreover, the significant results obtained remain a challenge to current scientific knowledge (Escolà‐Gascón, 2022a). It is said that epistemically, the hypotheses of RV are extraordinary because they have no rational or etiological foundations to explain the origin of these phenomena (Wooffitt, 2007). When an object of study is extraordinary (or implies anomalous phenomena), its scientific validation cannot be based on ordinary evidence (Tressoldi, 2011). However, the lack of epistemic foundations does not preclude or nullify the investigation of anomalous cognitions (see Cardeña, 2018; Hyman & Honorton, 2018). In fact, neither all scientific knowledge is rational, nor do all hypotheses under investigation have epistemic validity as noted by Henry (2005) and Leifer (2014). An example can be found in the mathematical theorems of incompleteness (Cheng, 2021; Visser, 2019), which demonstrate that, mathematically, the study or acceptance of undecidable questions, such as anomalous cognitions, does not imply rejecting rationality as the basis of scientific knowledge (see the current review by Kennedy, 2022). A clearer example is in the logical principle of *nonlocality* used in quantum mechanics (Mauri, 2021; Neppe & Close, 2015). If science accepts objects of inquiry that are extraordinary in questions of quantum physics and in mathematics, it at least should also be able to accept the scientific investigation (and not the scientific validity) of anomalous cognitions (Henry, 2005). We further contend that investigations of anomalous phenomena must adopt the principles of objectivity, confrontation, and the mutability of the scientific process (Bunge, 2013). Not applying this approach to the study of seemingly divergent or undecidable objects of study would otherwise result in the *Aristotelian fallacy of the negation of the consequent* and prevent the exercise of scientific falsification (Escolà‐Gascón, 2020a, 2020b). Moreover, assuming this conclusion without the contrast or application of the method would also have serious ethical consequences and promote scientific prejudice and pseudo‐skepticism that characterizes “scientism” (Houran & Bauer, 2022; Leiter, 2002; Truzzi, 1987).

### 1.4 The signaling theory of emotions

In his report, the former director of the SAIC RV research program mentions the role of emotions as a potential factor that could influence participants' performance (see May, 1996). The possible influence of emotions on RV testing was also mentioned in other subsequent publications (e.g., May & Marwaha, 2018). Recently, Escolà‐Gascón et al. (2022b) published with *Cell Press* a report on anomalous cognitions showing a quadratic relationship between the use of emotions and hits on precognition tests. Although the hits on precognition tests were unsuccessful, the significant relationship between perceived emotional intelligence (EI) and hits supports the possibility that EI may be an influential cognitive factor in the use of anomalous cognitions. One of the criticisms the authors received was that they measured perceived EI using self‐report questionnaires and not as a formal cognitive ability (see Escolà‐Gascón et al., 2022b). Therefore, one possibility for extending research on RV would be to include the assessment of EI as a cognitive attribute mediating the outcomes of anomalous cognitions. In the following paragraph, we propose a possible theoretical approach that could justify this association.

Salovey and Mayer (1990) developed a theoretical model of emotions and the meaning of EI. They viewed emotions as behaviors that emit signals with psychological meanings that are decoded by the receiving individuals or the environment (cf. Mayer & Geher, 1996). This decoding usually involves the activation of a rational‐strategic reasoning and cognitive reasoning based on intuition and experience (Mayer et al., 2000); both are grounded in dual models of cognitive processing (Evans, 2003; Osman, 2004). Similarly, the contents of decoding vary according to multiple factors ranging from sociocultural variables to more biological issues or individual differences (Mayer & Salovey, 1995). Within this model, EI is understood as a skill set to identify, discriminate, generate, and apply one's own emotions and those of others, as well as to use them for redirecting one's own thoughts or behaviors (Salovey & Mayer, 1990). Therefore, EI is not a personality trait but a cognitive attribute that is independent of the classical construct of general intelligence (Mayer et al., 2002). Mayer et al. (2016) created a cognitive assessment instrument (with hits and misses) to test EI, which was called the *Mayer—Salovey–Caruso Emotional Intelligence Test* (MSCEIT).

The rationale for linking EI to RV outcomes draws on the proposal that anomalous cognitions function as a crawl by an individual in search of distant information (May, 1996; Utts, 1995, 1996, 2018). In this case, the targets (e.g., the locations of places) that the RV participant must ascertain might—like emotions—have signals unknown to current scientific knowledge, yet detectable by certain people. An assumption of anomalous signals is based on the logical axiom of nonlocality (e.g., Lucadou et al., 2007); that they are detectable is the main hypothetical model tested here. Similarly, the *nonlocality* principle is also considered in MSCEIT indirectly; the original authors did not cite this principle in their theoretical justification, but it was deducible at the time that they employed the experiential and intuitive areas to measure EI.

More specifically, the signals that Mayer et al. (2016) attributed to emotions are not assumed to be a wave function equivalent to signals emitted by other physical systems (e.g., a cell phone antenna). The signal is a stimulus that contains key information (meanings); the stimulus or emotion is modeled as a signal because it communicates a message or state and not because the signal is a wave function. Understanding this point is vital, as anomalous cognitions also cannot be assumed to be physical signals measurable as wave functions. In fact, the targets used in RV are not rationally connected to sensory perception (through the conventional senses). The same is true for the meanings attributed to emotions (which remain undetermined until the individual makes an observation): the same emotion can have different meanings, and there is no logical chain of rational interpretations. For example, a person could interpret their experience of the “fear” emotion as feeling personally threatened. In the case of EI, the meaning of “feels threatened” is not exclusively the product of a logical‐strategic procedure, it also includes a dimension, that is, irrational and intuitive. This is the aspect that our study is interested in measuring.

### 1.5 The present study

Research on RV is useful and necessary for two essential reasons. First, it represents one of the frontiers of current knowledge. Science does not advance only by investigating what we already know; it must also confront uncertainty and transform the unknown into something operative and accessible to human knowledge (Leifer, 2014). *Second*, theorists currently lack knowledge of many of the regulating mechanisms of human perception and cognition (Khrennikov, 2015). Indeed, we should not exclude RV phenomena from the study of sensory and cognitive processes because there is evidence that indicates that anomalous cognitions ontologically represent more than methodological or statistical artifacts, perceptual disturbances, or clinical symptoms (Cardeña, 2018).

This study does not a priori affirm or deny the ontological existence of psi, instead the authors scrutinize anomalous phenomena in statistical and *falsificationist* terms (cf. Popper, 1959; Schooler et al., 2018). More concretely, we analyze differences between observed results and estimated expectations to verify the findings of the SAIC experiments as per Hyman's (1996) recommendations. Strictly speaking, any significant results would not validate the existence of anomalous processes in RV phenomena, but would strengthen the hypothesis in favor of psi‐related RV. Such an outcome would provide an important update on the status of these phenomena.

**FIGURE 1:**
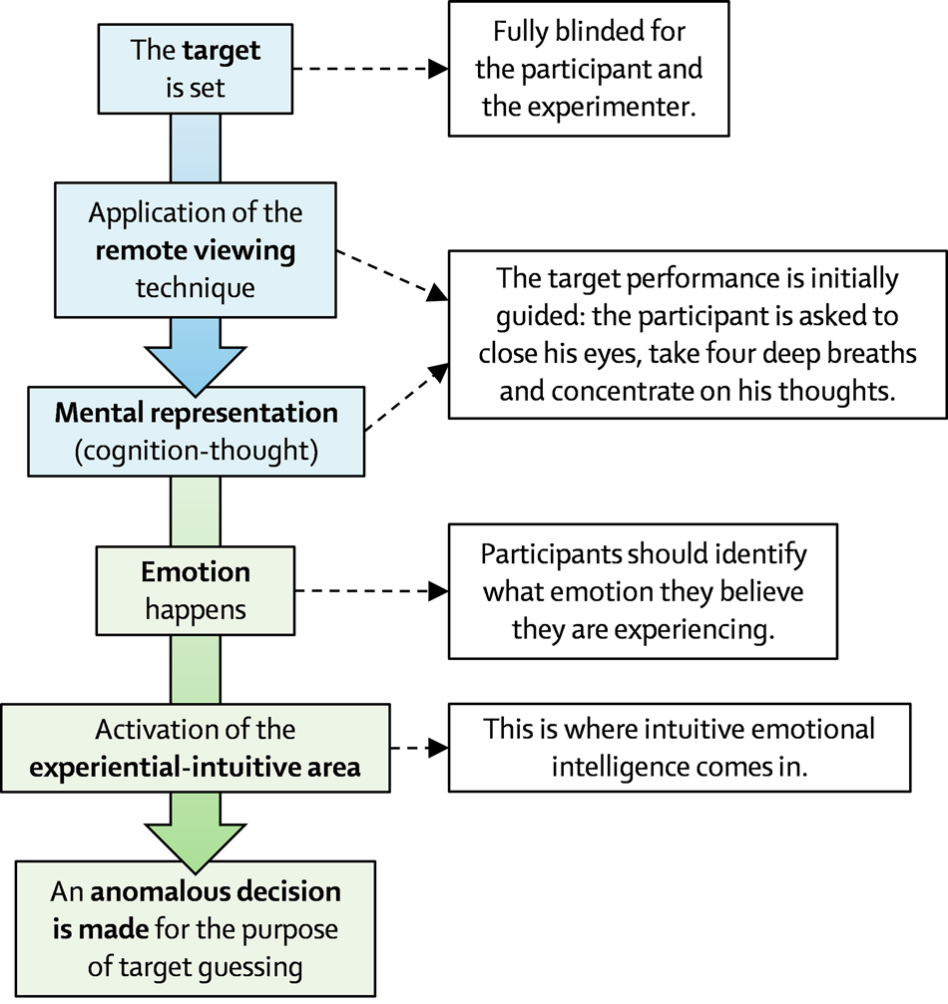
Hypothetical mechanistic model that relates emotional intelligence to the application of remote viewing. This figure also includes the logic of how the experiments were executed (see “Section 2” for more information).

Furthermore, the authors analyzed the association between experiential‐based emotional processes and RV outcomes—particularly, the relationship between the experiential area of EI and the participant's hit rate. If the targets were to function analogously to the experiential facet of EI, this would lend credence to the hypothesis that emotions play a key role in generating anomalous RV phenomena. The main difference with the MSCEIT model of EI is that in RV the strategic facet would not be used because there would be no sensory contact between the participant and the target. This would suggest the hypothetical model illustrated in Figure 1.

The model in Figure 1 is explained as follows: First, the target to be guessed is fixed (both for the coordinates and for the images). Next, the RV technique is used, and the participant is asked to visualize the type of place to which the target belongs. When applying RV, the participant is asked to close their eyes, take several deep breaths, and concentrate on their thoughts. Then, the participant activates their cognitive schemas and establishes an abstract thought‐representation of the supposed place. After this thought‐representation, an emotion should follow (this is based on the stimulus‐thought‐emotion‐behavior logic, see Lazarus, 1982). According to the dual process (see Evans, 2003; Osman, 2004) of EI as a cognition, the perceived emotion will be used by the participant as an experiential or intuitive procedure to make anomalous cognition decisions. Our exploratory hypothesis is to find out whether EI acts as a mediating variable between belief systems and psi test scores.

## 2 METHODS

### 2.1 Description of the sample

The sample consisted of 634 participants between 20 and 63 years of age (*M* = 41.25; *SD* = 12.45). Of these, 62% identified as women and 38% as men. All of them declared no prior psychiatric history and signed their informed consent to this research. Participants data were recorded anonymously.

The researchers formed two groups that had different experimental and sampling conditions: (a) *Group 1* consisted of people who reported no previous “psychic” experiences (nonbelievers, *n* = 347), and RV experiments based on coordinates of specific locations were applied (see Subsection 2.2.1. for more information); and (b) *Group 2* consisted of people who previously reported having “psychic” experiences (believers, *n* = 287), and RV experiments based on images of the locations identified by the coordinates were applied.

#### 2.1.1 Why participants were classified as “nonbelievers with coordinates” and “believers with photographs”

This classification and distribution of participants was based on previously published evidence found by other researchers. On one hand, the distinction between believers and nonbelievers was based on *sheep‐goat* effects, which show that experienced individuals have favorable attitudes toward parapsychology and perform better on experimental psi tests than nonbelievers (Thalbourne, 2001; Thalbourne & Houran, 2003; Thalbourne & Storm, 2012). This trend was recently observed in the replication by Escolà‐Gascón et al. (2022); although no significant effects in favor of anomalous cognitions were obtained, believing participants scored higher than nonbelievers on the RV tests.

On the other hand, CIA declassified reports from the SRI and SAIC revealed that participants tended to obtain better matches or hits when they applied RV with targets that were graphical representations (e.g., photographs). In fact, considering this pattern, May and Marwaha (2018) speculated that participants applying RV with photographs might be describing the characteristics of the contents of the photographs rather than the actual physical locations depicted in the photographs. If the previous evidence was correct, generating believer‐photographs and nonbeliever‐coordinate groups should maximize the observed statistical differences in scores between both.

Therefore, the criterion concerning why these two groups were formed, was supported by the previous statistical evidence, and we aimed to find out whether the previous evidence remained stable in the present replication.

### 2.2 Procedures and materials used

#### 2.2.1 Explanation and conditions of the new RV experiment

A RV experiment model was designed based on the techniques used in the SAIC, as well as forced‐choice designs. The interjudge design (applied in the original RV experiments) was discarded due to the associated methodological problems detected in the last decades and for being highly unstable (Kruth, 2021). Additionally, Hyman (1996) and other skeptical researchers criticized this design because the judges who evaluated participants' responses in the original experiments (determining to what degree participants' RV responses matched or not matched the targets) were not external to, or independent of, the SRI and SAIC research centers.

In our case, RV targets (i.e., targets to be guessed) corresponded to the locations of four types of places: (a) military bases, (b) hospitals, (c) schools (or education centers), and (d) cemeteries. The authors selected sites for their government interest and status as strategic locations in the event of conflict or outright war. Thirty‐two targets were registered (eight each of military bases, hospitals, schools, and cemeteries). The numbers were equivalent to ensure equiprobability of target type. The registration of the targets was applied via two means: (a) the geographical coordinates of their location were taken; and (b) exact images of the point indicating the coordinates were extracted from Google Maps. Even if the participants had no perceptual connection or access to the information of each target, this was important to evaluate whether the target's “presentation type” (i.e., *coordinate*‐based presentation versus *picture*‐based presentation) affected the experimental outcomes.

Each participant performed 32 trials: in each trial, one of the 32 locations was randomly selected beforehand. Specifically, the random selections were made taking into account the category of each of the locations: first, one location from each of the categories was randomly selected; second, after one location from each category had been randomly selected, one location category was also randomly selected from the four typologies. This chosen location is the one that the participant was expected to hit by supposedly employing anomalous cognitions. In the first random selection, there was replenishment of the locations for each trial; that is, after a location had been chosen from a specific category and for a specific trial, said location was available again to be randomly selected in the next trial. Participants were only informed that there were four types of locations and that they had to guess which of them had been previously selected. Participants in both groups also knew that in each selected category, a location was assigned.

For Group 1, each coordinate was printed on a micropaper that was stored in a small envelope, with this envelope then placed in an A5‐sized envelope (like matryoshka dolls). The envelopes were sealed, and both researchers and participants were blinded to their contents. An external technician assistant, independent of the researchers handled this process, and another support technician checked that the envelopes had no marks, transparencies, or otherwise showed evidence of tampering to ensure the internal validity of the protocol.

In each trial, the participant was shown an envelope containing the location coordinates of a place, which could not be opened. The participant could see the envelope but not physically touch it or manipulate it. The RV protocol was then implemented, participants were asked to close their eyes, take up to four deep breaths, and instructed to visualize, at least, to which type of place the randomly specified location within the two envelopes belonged. For up to 15 minutes, participants had to determine whether the target location was a military establishment, hospital, school, or cemetery. If the participant's choice matched the target category, +1 point (hit) was scored. When there was no match, 0 points were scored. At the end of each trial, although the correct answers were not shown to the participant, there was a margin of time for the participants to share with the experiment technician their first impressions. One month after the experiment, the participant could request to discuss their results with a researcher.

**FIGURE 2:**
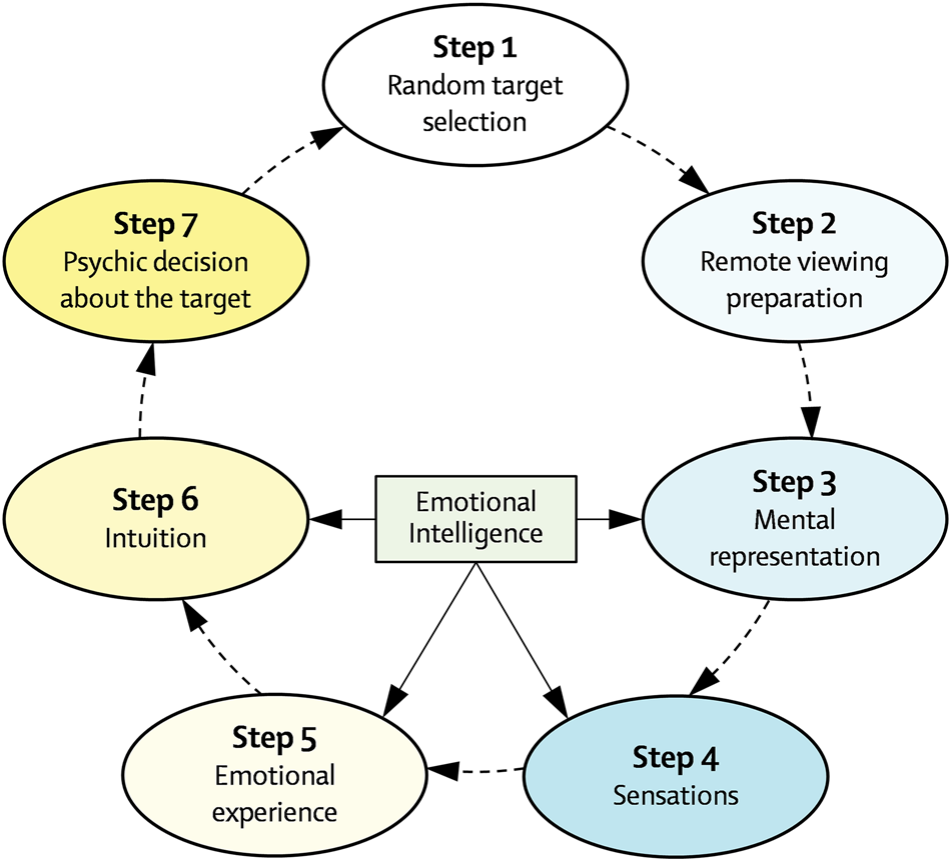
Graphical summary of the steps performed in the remote viewing experiment. These steps are in accordance with the proposed hypothesis in Figure 1.

For Group 2, the same envelope procedure used for concealing the coordinates of target locations was used to conceal the photographs of the target locations. The participants then followed the same trial procedure as Group 1, with the exception mentioned above. Finally, selection of the location in both the coordinates and photograph experiments was random and different for each trial and for each participant. Thus, the correction template or stimulus sequence was different across participants. Figure 2 summarizes in an operational manner and considering the contents of Figure 1, the steps of the experiments.

In total, 32 hits were possible, with an average of eight hits expected by chance (32/4 = 8). In each trial, the participant could also verbally describe the contents they individually visualized about the target location. This information was used for subsequent qualitative studies. The experimenter (vs. the study investigators) collated data and responses for each experiment.

#### 2.2.2 Specifications on the type of design used

In our research, we used the qualitative RV protocol originally employed by the researchers at the SAIC institute. However, if we were to use only these protocols, our study would be solely qualitative (with the limitations that this represents). To use quantitative measures, we included a forced‐choice design, in which the participant had to choose one of four specific alternatives (as explained in the previous subsection). Forced‐choice designs are more robust and valid than any other qualitative design. It is possible to combine the experimental tasks of the original RV protocols with the forced‐choice designs, generating a more complete and extended protocol than the original RV protocols.

For this reason, this research is a protocol replication of what the SAIC researchers did, but it is also an extension, as we integrate the forced‐choice protocols as outlined in the previous subsection. Clarifying this issue is crucial to avoid confusion and to better substantiate why we consider the present study a replication and also an improved extension of the original investigations that the CIA commissioned. By employing a forced‐choice design and quantifying the measurements, we can also employ more robust predictive models such as the structural equation models (SEM) that we explain in the statistical analysis subsection.

We hope to provide qualitative analyses in future reports; the present research focuses on the quantitative and forced version attributable to RV.

#### 2.2.3 Experimental controls

The controls for the experiment addressed the major methodological limitations of the SRI and SAIC experiments. Below outlines how the critical points highlighted by Utts (1995, 1996, 2018) were resolved in the present study:

1. One of the problems with the CIA‐funded SRI experiments was random selection of targets without replacement, such that, when a target was chosen, it was precluded from being chosen in the other trials. Utts (1995, 1996, 2018) and Hyman (1996) both noted that this practice could provide clues to the participant about the category to which the targets belonged. Thus, the design employed target replacement.
2. Another criticism of the SAIC experiments related to the coordinates of the targets. In the original experiments, the target's coordinates were shown to the participant; hence, participants knew the coordinates of the target that they were to describe. If any participant knew how to rationally interpret the coordinates of a function, this could reveal an approximate location and facilitate a guess. Accordingly, we concealed the coordinates from each participant using the envelope procedure, as outlined in Section 2.2.1., and participants could neither handle nor manipulate the envelopes.
3. Hyman (1996) noted that the lack of double‐blind conditions with the participants and researchers in the original experiments could have led to unwitting cuing of correct targets. Therefore, our participants had no contact with the researcher during the execution of the experiment. Instead, an experiment technician oversaw the protocol. Also, the technician and the participant were unaware of the random target selections. The computerized random selections and envelopes were prepared by an assistant independent of the experimenters and investigators, stored in a locked cabinet, and given to the experiment technician only at the time of the investigation. Additionally, the researcher had no contact with the independent assistant who made the random selections. This triple‐blind technique guaranteed methodological rigor with respect to conscious or unconscious cuing. Finally, participants did not have access to the computer that made the selections or to the envelopes with the coordinates.

### 2.3 The Mayer—Salovey–Caruso Emotional Intelligence Test (MSCEIT)

The MSCEIT was developed based on the model of dual information processing (Mayer et al., 2016). This measure consists of eight dimensions (or tests) that, when grouped together, are amenable to structural equation modeling of EI theory. These dimensions, and their associated activities and means of assessment, are: (a) *faces*—a task that determines whether the participant knows how to correctly recognize emotions in other people's faces; (b) *drawings*—an activity in which the participant must identify what emotions are being depicted in different representations of art, music, and activities in the environment; (c) *facilitation*—a cognitive task that examines the degree to which the participant is able to understand how moods influence behavior and thinking; (d) *sensations*—measures the degree to which the individual is able to correctly relate the emotions he or she feels to primitive sensations such as light, color, and temperature; (e) *changes*—assesses the degree to which the subject understands a chain of emotions and how emotions develop; (f) *combinations*—examines the participant's ability to classify and organize emotions into complex sets that define feelings; (g) *emotional management*—analyzes the individual's ability to employ their emotions and use them in decision‐making processes; and (h) *emotional relationship*—measures the same as the previous task, but instead of using their own emotions, the individual works with the emotions of others.

The scores for these six dimensions are converted to EI quotients (EQ) per respective normative groups. In this research, we used the sex‐differentiated normative groups belonging to the general Spanish population (see Mayer et al., 2016). These dimensions are grouped into four categories (i.e., perception, facilitation, comprehension, and management), which form two large “areas” (corresponding to the dual models of cognition): the *strategic area* (analytical type reasoning) and the *experiential area* (intuitive reasoning). Both areas collectively produce a total score of EI. The second‐order factors can be combined to form more summarized structures. For example, the eight dimensions can be used directly to estimate the two areas of intelligence that are of interest to our research, that is, the strategic and experiential areas (Mayer et al., 2003). Finally, we should emphasize that the reliability coefficients and internal consistency of MSCEIT scores in the present samples were acceptable across all dimensions (alpha coefficient > 0.8 and McDonald's omega > 0.8).

### 2.4 Sampling

**FIGURE 3:**
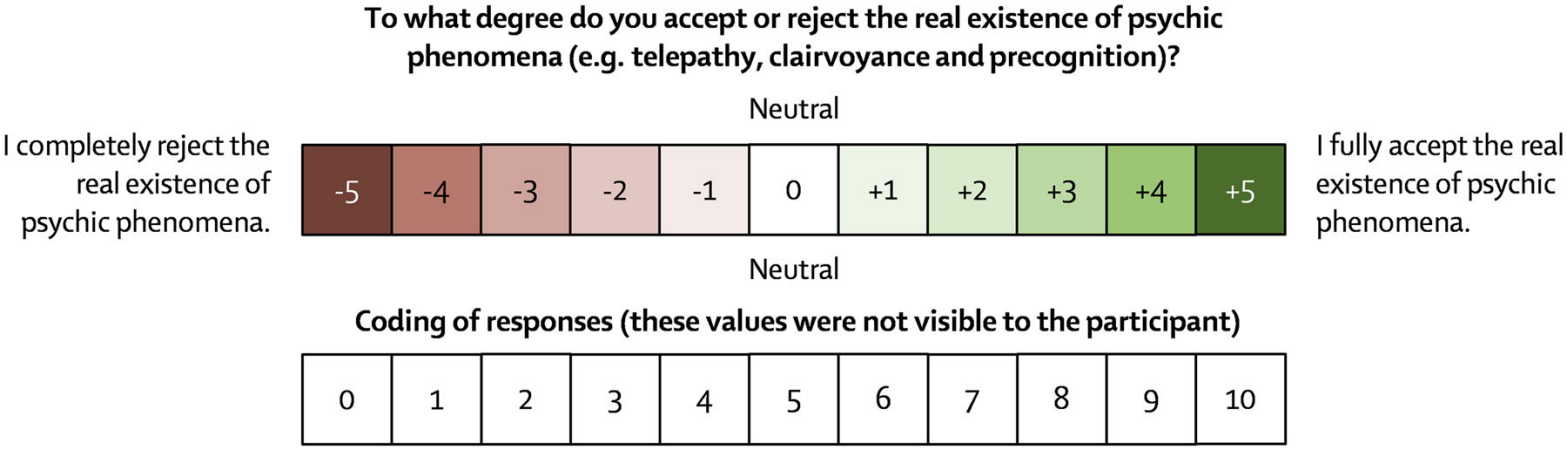
Example of semantic differential scale used in this study. Responses were coded from 0 to 10.

The sample selection was nonprobabilistic (meaning that participants were not chosen randomly). Participants were chosen from respondents to specific announcements in academic organizations (professional associations and colleges) and informal groups of believing individuals claiming to have had psychic experiences (these independent groups have a presence in social networks). Collaboration with these groups and organizations enabled the participation of the sample described in Subsection 2.1. Prior to the RV experiments, the participant was asked to respond to the MSCEIT and to specify, on a 10‐level semantic differential scale, their attitude toward parapsychology and psychic phenomena (see Figure 3).

Values or positions close to (−5) indicated a rejection of the possible existence of psychic phenomena and positions close to (+5) reflected an acceptance of the existence of such phenomena. Responses were coded from 0 to 10 to measure the degree of favorable attitude toward psychic phenomena. At the end of the MSCEIT and having answered this question, the participants were given a 15‐to‐20‐minute rest period before starting the RV experiment.

### 2.5 Statistical analysis

We processed the data with the *JASP* software (based on the R programming language, see JASP Team, 2023; The R Core Team, 2022), and the AMOS expansion of the SPSS statistical package was used for the SEM. Parameter estimation of the SEM analysis was based on the maximum likelihood criterion. This criterion was used to obtain a wide range of fit indices and to be able to perform the invariance analysis comparing Groups 1 and 2 (see Putnick & Bornstein, 2016). The invariance analysis is a method that allows us to know if the differences observed between the two groups are attributable to the conditions of the experiment or if, on the contrary, they are due to problems related to measurement bias.

Applied to the theoretical model of this study, this method has six levels of invariance that are set by establishing different restrictions: (a) configuration invariance (equality restrictions on the configuration of the theoretical model); (b) first‐order factorial invariance (equality restrictions are set on the factor loadings of the first‐order latent variables); (c) second‐order factorial invariance (equality restrictions are set on the effects or loadings of the second‐order latent variables); (d) scalar invariance (restrictions are set on the parameters related to the mediation effects involving the observed variables “hits” and “attitude toward psychic phenomena”); (e) residual invariance of the latent variables (equality restrictions are set on the latent variables receiving effects from others); and (f) residual invariance of the observable variables (equality restrictions are set on the errors attributed to the observable variables receiving effects).

Following Brown's (2015) criteria, we complied with at least the configuration invariance and the factorial invariance (although it is advisable to also comply with the scalar invariance in order to be able to carry out a contrast of the intercepts or latent means). The last two levels (i.e., residual invariance) are usually not fulfilled because the errors have a completely random statistical behavior. To check which levels of invariance are met and which are not, the changes or variability of three fit indices must be analyzed: the chi‐square statistic, the comparative fit index (CFI), and the root mean square error of approximation (RMSEA). In the case of chi‐square, the variation between the above levels should not be significant (*p* > .05). For the CFI and RMSEA, the variation should not be greater than 0.01 (Brown, 2015).

Analysis also determined whether participants' responses in the RV tests exceeded the expected statistical chance. For this purpose, a right‐handed one‐sided contrast was applied using the one‐tailed *t* test. We also calculated the Bayes Factor in favor of the alternative hypothesis (BF_10_) as an alternative estimator and set the a priori probabilities distributions at 50%; thus, there was equiprobability^1^ among alternative and null hypotheses. To avoid confusion, here we specify our statistical hypotheses: the null hypothesis was that the hits in RV experiments are not higher than expected chance; the alternative hypothesis (unilateral), is that the hits in RV experiments are higher than expected chance. The confidence level used in these analyses was 99% or higher.

## 3 RESULTS

**FIGURE 4:**
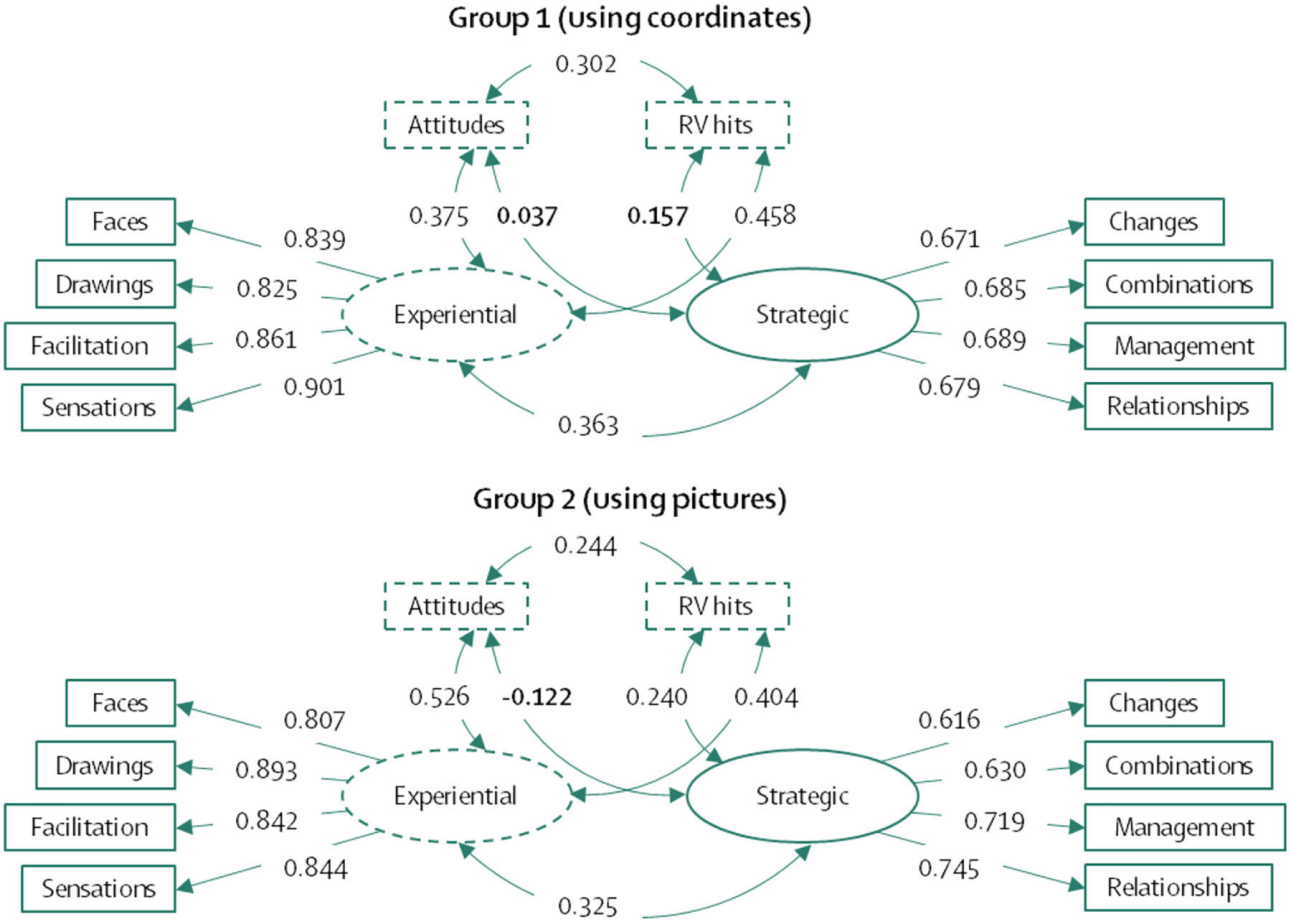
Correlational theoretical model that would justify the application of a fixed effects model (the model in Figure 5). Standardized effect parameters are shown with non‐significant parameters highlighted in bold. Discontinuous lines indicate a mediation effect between the variables “Attitudes” and “RV hits.”

**FIGURE 5:**
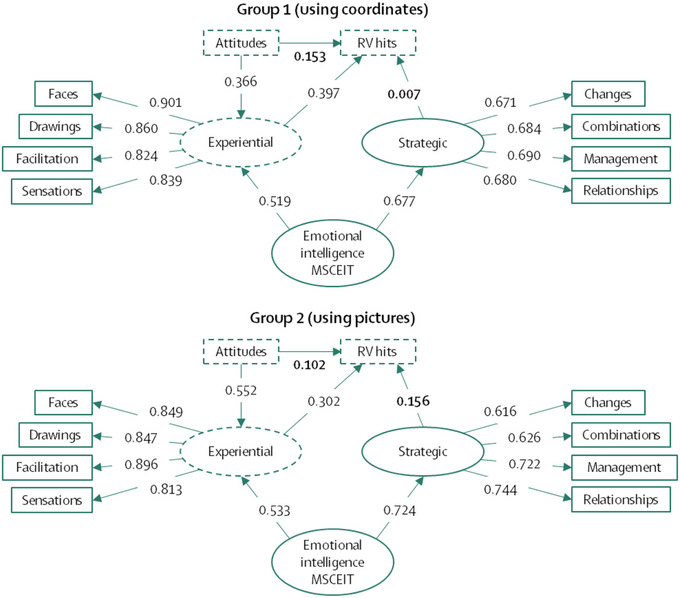
Theoretical models applied in groups 1 and 2 relating EI to hits in RV experiments. Standardized effect parameters are shown with nonsignificant parameters highlighted in bold. Discontinuous lines indicate a mediation effect between the variables “Attitudes” and “RV hits.”

Prior to the analysis of the contrast of means and the check as to whether the hits in the RV experiments exceeded the estimated chance, the authors wanted to analyze the theoretical validity relating EI to anomalous cognitions. In addition, we also wanted to statistically analyze whether the answers given to us by the participants and the scores obtained could be attributable to conditions related to the contents of the questions and the design of the experiment. If this were the case, there would be a bias problem in the MSCEIT and RV experiments. This analysis is carried out by studying the invariances that we explained in Subsection 2.4. Previously, Figure 4 showed the linear correlation models between the variables, which should allow the application of a mediation effects model among the variables. On the one hand, the fit indices for Group 1 were as follows: χ^2^ = 37.838; Normed χ^2^ = 1.221; RMSEA = 0.025 (0.001–0.050); AGFI (adjusted goodness of fit index) = 0.961; CFI = 0.995; TLI (Tucker–Lewis coefficient) = 0.993; IFI (incremental fit index) = 0.995; RFI (relative fit index) = 0.964; NFI (normed fit index) = 0.975. On the other hand, the fit indices for Group 2 were as follows: χ^2^ = 33.110; Normed χ^2^ = 1.068; RMSEA = 0.015 (0.000–0.071); AGFI = 0.962; CFI = 0.998; TLI = 0.997; IFI = 0.998; RFI = 0.962; NFI = 0.974. Due to the positive values of the fit indices in both groups, we were able to apply and analyze the fixed effects in Figure 5. Concretely, Figure 5 shows the theoretical models with the standardized parameter estimates (effects). Parameters that were not significant are bolded. These analyses were applied for both Groups 1 and 2. Similarly, invariance analysis was applied to the model in Figure 5.

The unmediated direct effects of “Attitudes” on “RV hits” were 0.302 (*p* < .001) for Group 1 and 0.244 (*p* < .001) for Group 2. As a first conclusion, we can infer that the mediation effects of the variable “Experiential” only reduced 15.1% 2 of the variance of the direct effects of Group 1 and 14.2% of the variance of Group 2. However, the effects of the “Experiential” variable on hits were significant; which allowed us to focus on the interpretation of these statistical effects.

The experiential area of the EI has small effects on the hits in the RV experiments. Although the effects are small (0.3 to 0.4), it was possible to calculate what proportion of the variance of the hits could be predicted by the experiential area: 15.8% for Group 1 and 9.1% for Group 2. However, in Group 2, the strategic area also contributed some information that, in total, predicts RV hits by 19.5%. In addition, the experience area acts as a mediating variable between attitudes towards psychic phenomena and RV hits. This observation complements the *Sheep‐Goat* effect, where greater belief in the paranormal positively correlates with greater sensitivity to internal and external stimuli (Thalbourne, 2001; Thalbourne & Houran, 2003; Thalbourne & Storm, 2012).

Table 1 shows the results of the invariance analysis. The goodness‐of‐fit indices provide insight to the theoretical validity of the model, as well as the presence or absence of bias in the responses and the design of the experiment.

**TABLE 1:** Analysis of the validity of the proposed theoretical model and analysis of invariance for bias control

Indices of goodness of fit	Invariance of configuration	First order factorial invariance	Second order factorial invariance	Scalar invariance	Residual invariance (latent variables)	Residual invariance (observable variables)
χ^2^	74.670 (*p* = .170)	87.238 (*p* = .107)	92.697 (*p* = .070)	95.946 (*p* = .061)	101.876 (*p* = .036)	126.601 (*p* = .004*)
Δχ^2^	–	12.568 (*p* = .128)	5.459 (*p* = .065)	3.249 (*p* = .197)	5.930 (*p* = .052)	24.725 (*p* = .003*)
Normed χ^2^	1.167	1.212	1.253	1.262	1.306	1.455
RMSEA (Threshold: < 0.05)	0.016 (∼0‐0.030)	0.018 (∼0–0.031)	0.020 (∼0–0.032)	0.020 (∼0–0.032)	0.022 (0.006–0.033	0.027 (0.016–0.037)
ΔRMSEA (Threshold < 0.01)	–	0.002	0.002	0	0.002	0.005
CFI (Threshold > 0.95)	0.996	0.994	0.993	0.993	0.991	0.985
ΔCFI (Threshold < 0.01)	–	0.002	0.001	0	0.002	0.006
AGFI (Threshold > 0.90)	0.961	0.960	0.959	0.958	0.958	0.953
TLI (Threshold > 0.95)	0.994	0.993	0.992	0.991	0.990	0.985
IFI (Threshold > 0.95)	0.996	0.994	0.993	0.993	0.991	0.985
RFI (Threshold > 0.95)	0.963	0.961	0.960	0.959	0.958	0.953
NFI (Threshold > 0.95)	0.973	0.969	0.967	0.966	0.964	0.955

*Note*. χ^2^, Chi square; AGF, adjusted goodness of fit index; CFI, Comparative Fit Index; IFI, incremental fit index; NFI, normed fit index, RFI, relative fit index; RMSEA, Root Mean Square Error of Approximation; TLI, Tucker‐Lewis coefficient .

All fit indices (including the chi‐square index, which is usually very sensitive in this type of analysis) supported the validity of the model in both Groups 1 and 2. This result indicates a robust relationship between EI and hits in the RV experiments. The invariance analysis was also positive, which suggests the residual invariance of the latent variables and all prior levels. This implies that there were no biases in the participants’ scores or in the design of the RV experiments.

Taken altogether, we conclude that the experiential area of EI clearly and positively influenced the hit rate in the RV responses documented here. We also surmise that no obvious biases altered and distorted the research outcomes. Table 2 begins the contrast of the mean values between Groups 1 and 2. Also included is the analysis of the latent mean of the EI score and the contrast of the intercepts of the SEMs in Figure 5.

**TABLE 2:** Descriptive statistics, latent means of factor scores, and intercepts

	Group 1 (using coordinate‐based targets, *n* = 347)		Group 2 (using image‐based targets, *n* = 287)		Latent mean of the factor scores of group 2 of the EI factor (setting the mean of group 1 to 0) and latent means of the intercepts assuming scalar invariance.	Student's *t*‐test (using Welch's correction) and Hedges' *g*‐tests
	Mean	Standard deviation	Mean	Standard deviation		
Hits	8.31	1.768	10.09	1.889	7.172 *p* < .001	−12.194** 0.975
Faces	98.12	17.45	105.95	16.479	85.380 *p* < .001	−5.798** 0.461
Drawings	99.08	17.712	105.43	17.142	86.026 *p* < .001	−4.567** 0.363
Facilitation	97.54	17.358	104.35	16.785	84.647 *p* < .001	−5.009** 0.399
Sensations	98.69	16.890	105.25	17.874	86.409 *p* < .001	−4.715** 0.377
Changes	100.55	17.901	104.32	16.984	102.247 *p* < .001	−2.715* 0.216
Combinations	98.79	17.142	106.31	16.911	102.135 *p* < .001	−5.539** 0.441
Emotional management	100.54	16.667	105.86	17.390	102.939 *p* < .001	−3.906** 0.312
Emotional relationships	99.21	17.305	105.81	16.417	102.328 *p* < .001	−4.915** 0.391
Strategic area	98.36	15.521	105.24	15.124	0 (equality restriction required)	−5.638** 0.449
Experiential area	99.77	13.341	105.58	13.025	0 (equality restriction required)	−5.522** 0.440
Total EI (MSCEIT)	99.07	11.688	105.41	11.279	3.619 (latent mean of factor scores) *p* < .001	−6.934** 0.552
Attitude towards psychic phenomena	3.71	1.752	5.94	1.937	It represents an observable variable of exogenous type; it has no intercepts or latent mean of the factor scores.	−15.099** 1.209

*Note*.

*p < .01 .

**p < .001.

EI, emotional intelligence.

The contrast of direct means between groups 1 and 2 is also included.

Analysis found significant differences and small‐to‐moderate effect sizes. In general, Group 2 scores exceeded those of Group 1. Specifically, RV hits increased in Group 2 by almost one *SD* over Group 1. The highest effects were found for the attitude toward psychic phenomena in Table 2. This increase in effect size could be explained by the fact that participants in Group 1 reported no prior psychic experiences, whereas those in Group 2 did. The same logic applies to increases in scores on the EI variables. The intercepts were clearly significant and represent the average value that would be obtained on the dependent variables when the value of “*x* = 0” in the function. Finally, the latent mean revealed that the Group 2 EI mean differed from the Group 1 mean by up to three *SD*s applied to the standardized factor scores (*z*). This is a more robust effect to consider rather than the direct difference observed for this variable. With these intercepts, this logic cannot be applied, as it requires setting the Group 1 intercepts to “0,” which would mathematically nullify the analysis because there is more than one “0” involved.

Table 3 provides the most important analysis of whether the hits were able to exceed the estimated mathematical expectation. Because these analyses are provocative to the skeptical approach of the authors, we wanted to include a division of the sample by systematically differentiating between participants with high levels of experiential EI and those with low levels. This differentiation was made according to two independent criteria: (a) we considered the original criteria based on the EI quotients of Mayer et al. (2016). In this case, scores equal to or above 110 would serve as a threshold to discriminate between highly competent participants, from those within the intervals of the mean (between 90 and 109 points) and against those with insufficient experiential EI levels (below 89 points). And (b), we also took into account the median of the EI levels of the “Experiential” dimension, which was 102. With these subdivisions of the total sample, we aimed to perform replications using a split‐sample approach to analyze the consistency of the results versus mere statistical significance (cf. Cohen, 1994; Dixon & Glover, 2020; Earp & Trafimow, 2015; Houran et al., 2018; Kornbrot et al., 2018; Tressoldi, 2012). We do not intend to replicate the contrast of the latent means because this is only a complement to analyze whether or not the average scores of the hits on the RV tests also exceed the expected chance in these new samples. In total, eight groups are presented: the first two were the two main groups analyzed above, the other three follow the criteria of Mayer et al. (2016), the next two were established according to the median and the last one provides the averages over the total sample.

**TABLE 3:** Analysis of the statistical significance of the hits in relation to what would be expected by chance

Group definition criteria	Groups	*t*‐test Unilateral contrast μ_Sample_ > μ_Exp. chance_	BF_10_ (error %) μ_Sample_ > μ_Exp. chance_	Descriptive and Gross differences regarding expected chance (≤8)	Effect sizes (Cohen's *d*‐criterion)	
					σ levels. (σ = 2.45)	*d* (μ = 8)
Nonpsychics, using coordinates as a target and lowest EI levels	Group 1 *N* = 347	3.25**	Bilateral = 10.428 (∼0%) Unilateral = 20.842 (∼0%)	Mean = 8.31 S.D. = 1.768 **Difference = 0.31**	0.31/2.45 = 0.126^a^	
Psychics, using pictures as a target and highest EI levels	Group 2 *N* = 287	18.8**	60.477 (∼0%)	Mean = 10.09 S.D. = 1.889 **Difference = 2.09**	2.09/2.45 = 0.853	
Criterion of Mayer et al. (2016). Participants with experiential EI ≥110	Group A *N* = 193	16.474**	44.837 (∼0%)	Mean = 9.79 S.D. = 1.507 **Difference = 1.79**	1.79/2.45 = 0.730	
Criterion of Mayer et al. (2016). Participants with experiential EI between 89–109	Group B *N* = 294	15.392**	45.291 (∼0%)	Mean = 9.62 S.D. = 1.807 **Difference = 1.62**	1.62/2.45 = 0.661	
Criterion of Mayer et al. (2016). Participants with experiential EI < 89	Group C *N* = 147	‐4.975 (p∼1)	0.015 (∼0%)	Mean = 7.22 S.D. = 1.890 **Difference = −0.78**	The contrast was unilateral and the mean observed was less than 8.	
Criterion according to the experiential EI median (>102)	Median Group 1 N = 312	20.925**	65.497 (∼0%)	Mean = 9.86 S.D. = 1.577 **Difference = 1.86**	1.86/2.45 = 0.759	
Criterion according to the experiential EI median (≤102)	Median Group 2 *N* = 322	3.040**	11.496 (∼0%)	Mean = 8.40 S.D. = 2.155 **Difference = 0.40**	0.40/2.45 = 0.163^a^	
All N participants	Total hits *N* = 634	13.9**	39.920 (∼0%)	Mean = 9.12 S.D. = 2.028 **Difference = 1.12**	1.12/2.45 = 0.457	

*Note*.

*p < .01.

**p < .001.

**Average effect size of SAIC experiments =** 0.447 (Average established only with significant effect sizes).

^a^ The effect size is less than 0.2, which is null and allows us to infer that these differences have no applied value and are not interpretable.

The average value expected by chance was 8 hits. The effect sizes considered the difference between this average value and the average total hits of each group and the limits based on the standard deviation. The theoretical standard deviation expected by chance was also used. This deviation was calculated as follows:

$$\sigma ∼\hat{\sigma }=\sqrt{32\times \frac{1}{4}\times \frac{3}{4}}=\sqrt{6}=2.45,$$

Therefore, the standard deviation, which is the average of the expected theoretical variability, was 2.45. Table 3 shows the comparisons between the means of the observed hits in each group and the theoretical mean expected by chance. Significant differences would indicate that the theoretical mean expected by chance was exceeded. Effect sizes would reveal the strength of the observed effect.

Group 2′s hit rate did significantly exceed chance expectations. In fact, the effect size of 0.853 is a comparatively high value given that the average effect size in the SAIC experiments was 0.447. This result—derived from the use of recommended improvements to the original protocols (cf. Hyman, 1996; Utts, 1995, 1996, 2018)—statistically suggests the presence of RV effect. In the remaining samples, the contrasts were significant in five of the eight samples. It should be noted that in groups A, B, and C, the significant contrasts coincide with significant increases in the experiential EI quotients. It is also true that in Group B, the experiential EI quotients were within the limits of normality, and the minimum effect size was 0.661, which is in line with what is suggested by the results of the previous SEMs. Finally, considering the significant results in the three groups that coincide with increases in EI levels, we have more statistical evidence that implicates the role of EI in producing RV hits.

### 3.1 On the thresholds according to expected randomness

Following classical logic in considering whether or not RV occurs, the average hits should be greater than 8. The crucial question here is how many hits greater than 8 are necessary to support the hypothesis that the anomalous cognitions have occurred. If we were to apply frequentist logic to a single person's responses, the most conservative threshold that the hits should exceed would be 10.45 hits (8 + 2.45 = 10.45). As the observed hits are discrete values, the value of 10 or 11 should be taken. This would be the case if we wanted to apply the rules of frequentist probability to the hits of a single person, but it is not the case when this threshold is applied to average values observed in different individuals and in different samples.

In our case, we are working with groups of people and, therefore, we use averages of hits with a margin of error or change. Specifically, the margin of variation of these averages is assumed to be the standardized average variability (i.e., the standard deviation). Therefore, upper and lower limits could be defined based on the mean ± the observed standard deviation, which would form the confidence interval. Confidence intervals represent the space of the most plausible probability of finding the observed mean. Therefore, an observed point mean would have two limits (minima and maxima), within which there would be fluctuations or average changes that would summarize all the hits of a particular group of individuals (within‐subject variability). The main implication of this is that it would not be entirely correct to apply the rule in the previous paragraph directly to the averages observed in each group. If the upper limit of the interval of the mean of expected hits by chance is 10.45, the comparative element should NOT be the observed mean as a point estimate (which in this case the highest would be 10.09, belonging to group two of Table 3), but should be the upper limit of the interval of the observed mean (which would be 10.09 + 1.889 = 11.979). Therefore, the comparison between the direct observed mean (10.09) and the average upper limit of what is expected by chance (10.45) is not appropriate. The comparisons should be made at the same level of inference and, consequently, we obtain that 11.979 is more than 10.45. We reassert that this would not be applicable to the total hits of a single case; as we are analyzing sets of cases and samples, we must take into account such average variations based on standard deviations and attributable to the observed mean.

Finally, the evidence (11.979 > 10.45) allows us to conclude that in this study certain significant results were obtained in favor of RV. Moreover, considering the sample characteristics of this group (high EI and favorable attitudes toward experiencing anomalous cognitions), we have further reason to infer that these are favorable sample conditions for openers in RV tests.

## 4 DISCUSSION

Our research had two objectives: (a) to test RV in quasi‐experimental conditions and in an updated manner, following the proposals of the research initially commissioned by the CIA and conducted at SRI/SAIC; and (b) to seek an alternative approach to the affirmation‐denial dichotomy on whether RV effects are scientifically verified. We, therefore, divide our commentary into two parts. One section proposes our interpretations and implications of the results, and the other addresses the question of whether RV phenomena are scientifically established.

### 4.1 The use of EI in anomalous cognitions

The SEMs in Figure 4 and the fit indices strongly suggest a valid link between EI and RV hit rate. Of course, these correlations did not correspond to very strong effects and so should be interpreted with caution. We suspect here that EI is primary; that is, higher experiential EI leads to higher RV hit rates. An analogous hypothetical interpretation is that increasing the levels of EI also increases the likelihood of correct RV “guesses.” The difference between the first interpretation and the second is in methodology. Outside the purely experimental realm, yes, we can say that EI levels influence increases in RV hit rate. However, if we consider the strict application of the experimental methodology, the above affectations could not be stated in causal terms because there was no random assignment of the participants to the experimental conditions.^3^ Within the framework of statistical (and not empirical) causality, we can consider the fixed effects of the exogenous EI variable on the RV hit rate (endogenous variable) as statistically occurring. This means that, within the statistical framework, at least, increases in RV effects do occur when EI increases. This link allows us to explore what role emotions play in the production of anomalous cognitions. The following section outlines one speculative process model that should be tested in future research.

#### 4.1.1 The emotional Production‐Identification‐Comprehension (PIC) model for anomalous cognitions

Much research outside the RV literature indicates that emotions play an essential role in the production of behaviors (Lazarus, 1982). The ABC behavioral model of psychology (Antecedent‐Behavior‐Consequence: Iwata et al., 1994) asserts that emotion is a response or a consequence of thought, which is preceded by an antecedent stimulus (Zajonc, 1980). Emotion promotes consequently other behaviors or responses that become part of the ABC loop, interacting with other stimuli and restarting the whole process chain. The ABC model could be applicable in the case of RV, if we include emotions as one of the most essential variables in this process. While other behaviors do not require emotions to be executed, some highly complex behaviors do require emotional perception. In these situations, emotions act as precipitating factors or “precursors” of the behavior. We believe that something similar might happen with psi‐related functioning.

The stimulus would be the target that the participants must perceive or ascertain, the thought would be the cognitive reasoning that the participant establishes to mentally represent the target (the RV technique is applied here). The cognitive reasoning and mental representation would have an emotional impact on the participant. Upon perceiving an emotion (or, even simply, a sensation), the participant connects with the mental representation and makes a cognitive judgment. This judgment is a consequent or behavior that might correspond to the hit‐miss results in the RV experiments. Within this context, it seems plausible that individuals with high emotion production, identification, and understanding can more effectively leverage their emotional reaction to find the correct response in RV experiments. Indeed, in everyday life, the functional use of emotions has been shown to be a decisive factor in behavioral modifications (Brackett et al., 2004). These reasons collectively lead us to posit that individuals with high EI should exhibit higher hit rates on RV tests (and perhaps other types of psi‐related experiments or outcomes).

By way of further explanation, within the stimulus‐thought‐emotion‐response loop, the part that interests us most in this research is emotion. If we pay attention to the parameter estimates in Figure 5, specifically in the experiential area variable, we observe that the *strategic area* predicts very little variance in RV hit rate (in fact, these parameters were nonsignificant). The fact that only the *experiential area* of the MSCEIT is significant implicates emotional processing in the production of anomalous cognitions. Consequently, the statement in the previous paragraph could be modified as follows—*individuals capable of producing or processing emotions with ease, that is, know how to identify them and their meanings, will be those who perform better on RV tasks*.

This hypothetical process is called the “*Production‐Identification‐Comprehension* (PIC) Model.” It predicts that RV hit rates should be modifiable if we assume it is possible to train individuals to increase their EI abilities. However, PIC is for now only a statistically (and not empirically) valid model, which means that it will be necessary to apply it in further research and to investigate it strictly under experimental conditions. That said, our interpretations and proposals seem to agree with independent research showing that people with higher levels of transliminality (or the similar constructs of thin mental boundary functioning or heightened sensory processing sensitivity) also score higher on various measures of putative psi (Thalbourne & Houran, 2003; Thalbourne & Storm, 2012; Ventola et al., 2019).

#### 4.1.2 PIC as both a complement and uncertainty

The findings inherent to the PIC Model represent a crucial corroboration of previous research correlating alterations in consciousness with anomalous cognitions (e.g., Krippner et al., 1972; Luke, 2011). When consciousness does not remain in its “ordinary” state, it produces emotional responses that can interact with the contents of phenomenology of trance states (Polito et al., 2010). A similar analogy could be made with so‐called “haunt or poltergeist” episodes, which are related to psychophysiological “dis‐ease” states (e.g., Laythe et al., 2021). We do not intend here to explain the theoretical basis of altered states of consciousness but merely emphasize that our results align to previous evidence, and, for this reason, the PIC framework complements prior findings and insights about psi‐related experiences. We even suggest that the negative correlations that Escolà‐Gascón (2022a) found between the results of RV experiments and altered states of consciousness might be due to the difficulty of applying EI in trance states and, consequently, could also be along the same lines as this proposal. Indeed, Utts (1995, 1996, 2018) likewise emphasized that it is easier to find participants who can easily produce anomalous cognitions than it is to train them (cf. Tart, 1976). This assertion may well be correct, in that it is only EI (specifically PIC) that would be trainable versus psi‐functioning. Future research should explore whether PIC is a trainable component and, thus, a possible catalyst for anomalous phenomena.

However, we acknowledge the uncertainties of the proposed PIC Model. The nonlocality hypothesis still holds in the PIC model for several reasons. This study did not address which connector allows for the relation or translation of emotions to anomalous cognitions. Similarly, because of the speed at which the stimulus‐thought‐emotion‐response cycle occurs, we also do not know to what degree emotional production (and not mental representation) is the precipitating factor in successful RV responses. Moreover, that emotions are related to RV does not mean that this correlation is stable with the other anomalous phenomena we highlighted in the introduction. Therefore, this reinforces the need for additional studies on anomalous cognitions, and specifically on identifying the underlying mechanisms for these types of phenomena.

### 4.2 Are the anomalous phenomena scientifically established?

Starting in 1995 and after declassification, the American Congress, through the organizations that had developed the experiments on RV, commissioned Professors Hyman (1996) from the University of Oregon and Utts (1995, 1996, 2018) from the University of California to prepare a review report on the results obtained in the research programs that the CIA originally funded and conducted. Reviews should answer the question of whether “psi” phenomena are scientifically established. However, the expression “being scientifically established” (the original expression used in the reviews by Utts and Hyman) can have at least two meanings that would not be mutually exclusive but do have logically conflicting features.

On the one hand, the expression could be interpreted exclusively from a statistical or probabilistic judgment. In fact, the approach and statistical judgment used by SRI and SAIC consisted of the application of hypothesis testing based on statistical scrutiny. Specifically, these tests analyzed the statistical significance of the discrepancies between the observed measurements (obtained in the trials and experiments) and the estimated mathematical expectation (see the *Mathematics Handbook* published by Escolà‐Gascón, 2022c for a major revision). Consequently, this kind of statistical judgment would entail interpreting the occurrence of a given phenomenon as a set of significant deviations that may be above or below the estimated mathematical expectation. This probability inference would make it possible to ensure that the measurements of the deviations are not explained by the set of random (or chance) fluctuations.

However, this interpretation does not allow empirical assurance of when the supposed measured phenomenon is occurring (Escolà‐Gascón, 2022a, 2022b). Therefore, within the statistical‐probabilistic approach, concluding that a phenomenon is “scientifically established” should mean that only sufficient significant deviations were obtained (quantified by effect size tests), which were consistent and stable in relation to their measurements. If we focus on this approach, the conclusion that a phenomenon happens consistently and is statistically stable should not imply acknowledging or admitting that such a phenomenon is empirically real. However, the fact that the deviations are significant and are not explained by random fluctuations does represent statistical evidence supporting the hypothesis associated with RV.

On the other hand, in science, from a strictly factual approach, when an object of study is “scientifically established,” it means that sufficient evidence has been obtained to justify the real and functional existence of that object of study. Given the justification based on the burden of proof (or proofs), the object is formally accepted and established within the corpus of scientific knowledge. Unlike the probabilistic and statistical approach, empirical scrutiny would allow us to specify when a given phenomenon does or does not occur (if the scrutiny complies with experimental conditions and controls). These two interpretations based on different paradigms or approaches are crucial to an accurate understanding of the conclusions of the theoretical evaluations presented by the two professors cited above. The question that arises from these two interpretations is: can we consider that Jessica Utts' judgment was centered on the first interpretation and Ray Hyman's on the second? If so, both professors would be correct in their conclusions because they used different perspectives on scientific inference.

From a thorough review of declassified SRI and SAIC reports and publications, Utts (1995, 2018). concluded that anomalous phenomena (or psi‐functioning) were scientifically established. She also argued that the scientific challenge would not be in rereplicating the SRI and SAIC experiments, but in conducting research that would address the underlying mechanisms involved in producing the anomalous phenomena. An important note here is that Utts acknowledged the methodological limitations with the SRI experiments and explained how these were remedied in experiments subsequently conducted at SAIC. Utts’ statistical and methodological explanation suggests that her conclusion refers to the statistical (versus empirical) approach. In the same vein, Utts did not mention the word “empirical” and does not use expressions referring to possible evidence beyond the statistical judgment itself. Therefore, her conclusions based on effect sizes of deviations should not be incorrect if taken within the framework of statistical scrutiny.

In contrast, Hyman (1996) concluded that there was insufficient evidence to accept RV as a scientifically established phenomenon. He criticized that, for a phenomenon to occur, it is not necessary to resort to estimated mathematical hope (i.e., chance). His argument referenced the phenomenon relative to the psychophysical study of memory. This suggests that Hyman interpreted Utts' conclusions from an empirical and not a statistical approach, which could explain why there were so many discrepancies between the two authors' assessments. Furthermore, we must also bear in mind that not all phenomena are empirically observable and, consequently, only mathematical representation and statistical judgment would be scientifically available in decision‐making (Escolà‐Gascón, 2022c). Many phenomena have no direct observation in the physical sciences (e.g., the state of temperature and variations over time). In this sense, the fact that a phenomenon is not empirically observable and recordable does not make it a “pseudoscientific concept” (i.e., that it does not have sufficient epistemic foundations, see e.g., Fasce et al., 2021).

There is another essential nuance in that both professors agreed on several points and interpretations. Here, we will highlight the main agreement, as it is one of the reasons supporting a replication such as the present study. Hyman and Utts concurred that the significant effect sizes of the multiple SAIC experiments were statistically consistent or very similar to each other. Likewise, Hyman added that these nonrandomly attributable coincidences were not conclusive in themselves and that, only with further research replications could obtain more information on whether these sizes remain stable. This means that new replications should be carried out with the maximum conditions of experimental control and rigor. Ultimately, both evaluation reports provided helpful appraisals of the scientific value of the CIA and DIA's RV experiments. However, our narrative analysis suggests that both Utts and Hyman were correct from empirical versus statistical points of view and that their contributions, thus, have different impacts and implications.

#### 4.2.1 Do our results show statistical evidence of an anomalous effect?

Table 3 shows some effect sizes with provocative implications. To clarify, the effect size indicates the degree to which the “hits” (i.e., correct responses or information) exceeded chance expectations. The most relevant results to highlight at this point reference mediation effects and involve Group 2 (high levels of experiential EI, Cohen's *d* = 0.853), Group A (high levels of experiential EI, Cohen's *d* = 0.730), Group B (moderate levels of experiential EI, Cohen's *d* = 0.661), and Group 1 (high levels of experiential EI, Cohen's *d* = 0.759). The total effect size (including the 634 cases) was 0.457.

There are two criteria for interpreting these results. First, we could use the classic Cohen (1988) criterion. This is rather arbitrary, but it continues to be widely used and accepted as valid. Cohen (1988) suggested that values below 0.20 indicate no effect; between 0.21 and 0.49, the effects are small; between 0.50 and 0.70, the effects are moderate; and values greater than 0.70 are large effects. Applying these criteria to Table 3, we find that those groups with high scores on EI showed large effects. The effect sizes likewise decrease as EI decreases in the groups. We lack sufficient data for a correlational analysis but can tentatively confirm this trend via visual inspection, which certainly should be tested in new and future research. As Truzzi (1987) suggested, extraordinary objects of study require analyses and interpretations that go beyond the canonical.

Second, we could apply Ferguson's (2009) statistical criterion. Unlike Cohen's (1988), Ferguson's (2009) approach is based on what effect sizes should be obtained in order to be able to make consistent statistical inferences. Following this principle, a minimum value of 0.4 is needed to assume a small effect. Values equal to or greater than 1.15 indicate moderate effects, and those above 2.70 are strong effects. Using these thresholds, our results can be interpreted as small rather than moderate or large. This implies a lower level of consistency of the inferences, and therefore more original research is needed to make firm conclusions.

However, a critical point is that effect sizes are only minimally acceptable (greater than 0.4) when individuals score high on EI. This coincidence and the significant differences obtained with SEM analyses of invariance do support a possible direction of scientific research regarding the explanation of why anomalous cognitions occur—that is, it is necessary to understand the role of emotions and how participants manage them (per the level of EI). This does not mean to defend that EI is real or not real; we simply propose that, in the same way that there are skills (referred to as intelligence) that allow us to regulate certain decisions and actions, these skills could also be applicable to the regulation and use of emotions. We strive to address this point in our RV research.

Taken altogether, we contend that our results certainly constitute “statistical anomalies,” as they clearly defy the expectations of probability theory. Along these lines, it is crucial to assess to what degree these statistical anomalies are evidence for anomalous cognition. An anomaly represents just that: something strange that should not happen in statistical terms but does occur. And this occurrence is not one‐off, because similar observations are documented across other, independent studies that we previously cited. Such findings do not equate to explanations, so they do not establish the ontological reality of putative psi. That said, we must concede that the effect sizes of these statistical anomalies are consistent with the hypothesis that human cognition is not limited to known scientific knowledge and orthodox theories. Our results certainly highlight that the hypothesis proposal of the first scientists to address RV is not necessarily incompatible with scientific knowledge (see e.g., *Nature* publications Targ & Puthoff, 1974; Tart et al., 1980). Nevertheless, the statistical anomalies observed here and elsewhere add to the growing body of empirical literature that justifies continued research in this area of consciousness studies.

### 4.3 Limitations and conclusions

Although the preceding discussion highlighted major limitations of our study, arguably the most relevant of these to consider in future research are: (a) the methodology was quasi‐experimental versus strictly experimental, which limits causal statements; (b) the positive and significant association between EI and RV hit rates does not imply that emotions are necessarily the underlying mechanism for RV effects; and (c) following Hyman (1996), Group 2′s above‐chance scoring only implies a statistical versus empirical verification of RV phenomena. We should also underscore that our study was not preregistered, so new research should be conducted in ways that can be externally verified. Describing hypotheses, methods, and analyses before a study is conducted helps to foster transparency and, thus, reduce publication bias, especially with respect to controversial topics like RV phenomena (for a discussion, see Rabeyron, 2020).

Therefore, this updated report on RV and the experiments commissioned by the CIA and DIA allow us to state the following: (a) RV experiments (investigated under RV conditions and discarding the survival hypothesis) yield above‐chance results. (b) The fact that statistical chance has been overcome does not empirically validate RV but rather provides statistical verification of a robust anomaly that suggests anomalous cognition might be ontologically “real.” (c) EI and specifically PIC skills significantly predict RV scores between 9 and 19.5%. This raises the possibility that emotions could directly or indirectly precipitate anomalous cognitions (and perhaps even other psi‐related cognitions). (d) Anomalous cognitions should only be regarded as scientifically established phenomena within statistical and mathematical contexts but not be accepted as empirically validated phenomena due to the lack of tangential evidence causally linking physical mechanisms to the observed effects.

Finally, our previous publications have echoed Hyman's (1986) skepticism about the ontological reality of psi (e.g., Dagnall et al., 2016; Drinkwater et al., 2021; Escolà‐Gascón, 2020a, 2020b; Houran et al., 2017, 2018; Irwin et al., 2012a, 2012b; Lange et al., 2019). But we also defend the principles of neutrality, intellectual humility, and falsification in scientific research. Thus, the present results compel the authors to voice an updated position statement, that is, *our skeptically oriented team obtained ample evidence supporting the existence of robust statistical anomalies that currently lack an adequate scientific explanation and therefore are consistent with the hypothesis of psi*. This outcome stands in stark contrast to the literature on experimenter and observer effects, which are often cited as substantial hindrances to psi effects (Kennedy, 2003). Our findings certainly undermine this view as a blanket statement. We accordingly recommend that new studies both welcome and leverage the participation of proper skeptics in “adversarial collaborations.” These exercises are rarely used in parapsychology but involve researchers with differing views who jointly construct and implement studies that fairly address controversial issues while controlling for obvious ideological biases or methodological artifacts (e.g., Hyman & Honorton, 2018; Lange et al., 2004; Laythe & Houran, 2022; LeBel et al., 2022; Schlitz et al., 2006). Indeed, we agree with Cowan et al.’s (2020) assertion that this approach might be the most productive way to change current scientific views on highly controversial topics.

### PEER REVIEW

The peer review history for this article is available at https://publons.com/publon/10.1002/brb3.3026.

## Data Availability

The raw data supporting the conclusions of this article will be made available by the authors, without undue reservation.

## ENDNOTES

1 The a priori distributions that were adjusted were the default distributions configured in the JASP software (based on the R programming language). These a priori distributions are set such that the probability that the null hypothesis is true is 50%, and the probability that the alternative hypothesis is true is also 50%. These a priori probabilities (based on the default a priori distributions) are configured in this way when a neutral position is assumed with respect to the certainty of the hypotheses. This means that one does not pretend to be either for or against any hypothesis. For the default a priori distributions in JASP, the reader and reviewers are referred to Heo et al. (2020).

2 The proportions of variance explained are obtained from the linear combination of the squared observed standardized effects. This value can be interpreted as the amount of fluctuations (or variance) that an independent variable (or exogenous variable) predicts in another variable considered dependent or endogenous (see Brown, 2015).

3 Because no strictly experimental controls were applied, we must also highlight the hypothesis that individuals with RV learned to have more EI and, therefore, psi test successes should also affect EI scores. We acknowledge that this could be a possibility, but as a hypothetical interpretation, it is weak because we cannot scientifically verify which individuals have psi and which do not. To be sure which individuals have psi and which do not implies accepting that RV is a real cognition and this has serious implications for research. We (the authors of this manuscript) do not accept a priori that psi is real, and the hypothetical model posed in the introduction is *falsificationist* (not *verificationist*), which precludes applying this hypothetical interpretation to the findings.
